# Japanese Nationwide PCI (J-PCI) Registry Annual Report 2019: patient demographics and in-hospital outcomes

**DOI:** 10.1007/s12928-021-00832-0

**Published:** 2022-01-12

**Authors:** Hirohiko Ando, Kyohei Yamaji, Shun Kohsaka, Hideki Ishii, Hideki Wada, Sumio Yamada, Mitsuaki Sawano, Taku Inohara, Yohei Numasawa, Yuji Ikari, Tetsuya Amano, the J-PCI Registry Investigators

**Affiliations:** 1 Department of Cardiology, Aichi Medical University, 1-1, Yazakokarimata, Nagakute, Aichi 480-1195 Japan; 2 Department of Cardiovascular Medicine, Kyoto University Graduate School of Medicine, Kyoto, Japan; 3 Department of Cardiology, Keio University School of Medicine, Tokyo, Japan; 4 Department of Cardiovascular Medicine, Gunma University Graduate School of Medicine, Gunma, Japan; 5 Department of Cardiovascular Medicine, Juntendo University Shizuoka Hospital, Izunokuni, Japan; 6 Department of Health Sciences, Nagoya University Graduate School of Medicine, Nagoya, Japan; 7 Department of Cardiology Assistant Researcher, Tokyo Dental College Ichikawa General Hospital, Tokyo, Japan; 8 Department of Cardiology, Japanese Red Cross Ashikaga Hospital, Ashikaga, Japan; 9 Department of Cardiology, Tokai University School of Medicine, Kanagawa, Japan

The Japanese Percutaneous Coronary Intervention (J-PCI) registry is endorsed by the Japanese Association of Cardiovascular Intervention and Therapeutics (CVIT) and is designed to provide basic statistics on the performance of percutaneous coronary interventions (PCI) at nationwide level in Japan [1]. The primary objective of the J-PCI registry is defined as follows:

1. To collect reliable data and accurately describe the entirety of PCIs performed within country.
2. Seek for unmet needs regarding PCI, and further conduct in-depth analysis to find potential solutions.
3. Perform consecutive case registration as a prerequisite for institution certification, and audit data regularly to ensure its accuracy.

As of 2020, more than 200,000 PCI cases are registered annually from approximately 900 facilities that account for more than 90% of PCI-performing hospitals. Registration to the J-PCI registry is a prerequisite for certification as coronary interventionalists or educational institutions via CVIT. The accuracy of registered data is maintained by data auditing (10–20 institutions annually), which is conducted by members of the CVIT Registry Steering Subcommittee. The actual operation of the registry is managed by the CVIT Registry Academic Committee (chair: Tetsuya Amano, Aichi Medical University), which was established in the summer of 2018. Summary of the accumulated data is presented annually at the annual CVIT congress and are featured on the CVIT website (http://www.cvit.jp/registry/annual-report.html). In the present report, we report the analyzed patient-level data from the J-PCI registry between January and December 2019, comparing it with data from the previous years. Definitions of key baseline variables and categories upon clinical presentation can be found in the Supplementary Appendix.

## Number and age of registered patients

**Fig. 1:**
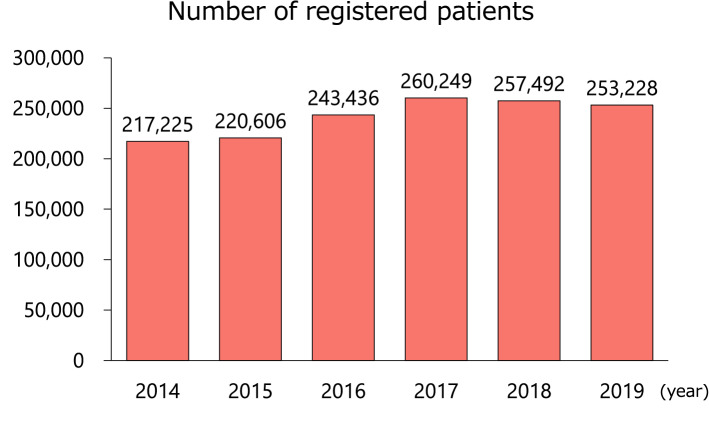
Number of registered patients. The annual numbers of patients registered in J-PCI over the past 6 years are shown. J-PCI, The Japanese Percutaneous Coronary Intervention

In 2019, 253,228 patients who underwent PCI were registered in the database (Fig. 1). The number of registered patients has remained largely constant since 2016. The mean age of the patients was 71 years, an increase of about 1 year over the past 6 years, probably reflecting the aging of the Japanese population (Supplementary Fig. 1).

## Patient characteristics

**Fig. 2:**
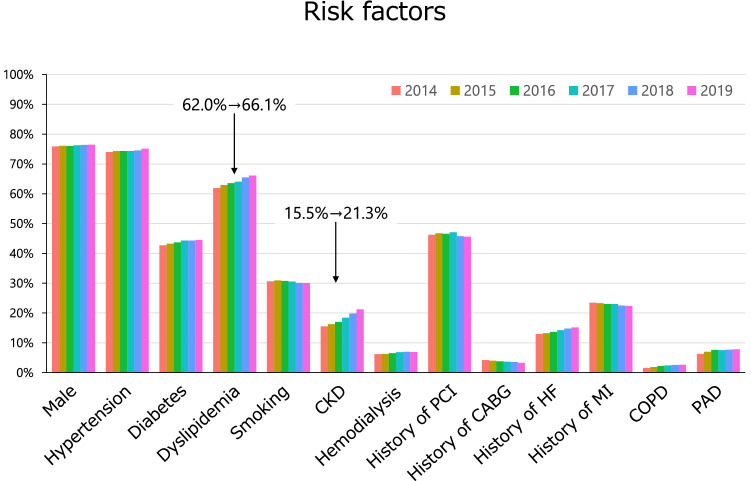
Prevalence of risk factors. The prevalence of risk factors over the past 6 years is shown. CKD, chronic kidney disease; PCI, percutaneous coronary intervention; CABG, coronary artery bypass grafting; HF, heart failure; MI, myocardial infarction; COPD, chronic obstructive pulmonary disease; PAD, peripheral artery disease

**Fig. 3:**
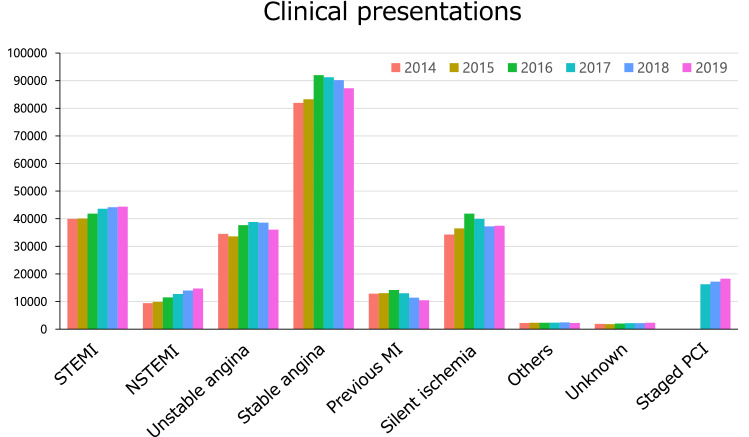
Number of patients with various clinical presentations. The annual numbers of patients with various clinical presentations over the past 6 years are shown. STEMI, ST-elevation myocardial infarction; NSTEMI, non-ST-elevation myocardial infarction; MI, myocardial infarction; PCI, percutaneous coronary intervention

The prevalence of patients’ risk factors is shown in Fig. 2 and Supplementary Fig. 2. The prevalence of dyslipidemia was gradually increasing over the past six years, and the prevalence of chronic kidney disease was also increasing. As clinical presentations, 95,063 patients (37.6%) underwent PCI for acute coronary syndrome including ST-elevation myocardial infarction (STEMI, *n* = 44,348), non-ST-elevation myocardial infarction (NSTEMI, *n* = 14,696), and unstable angina (*n* = 36,019) (Fig. 3 and Supplementary Fig. 3). Over the last 6 years, there has been a slight upward trend in the number of patients with STEMI or NSTEMI, while stable angina has been on the decline since 2016. In terms of preprocedural cardiac testing for myocardial ischemia in patients with stable angina, invasive fractional flow reserve measurement was added to the J-PCI registry since 2017 and has been gradually increasing since then (Supplementary Fig. 4).

## In-Hospital outcomes

The recorded in-hospital clinical outcomes include the following variables within the J-PCI registry: in-hospital mortality, postprocedural myocardial infarction, cardiac tamponade, acute heart failure/cardiogenic shock, stent thrombosis, access/non-access site bleeding events requiring red blood cell transfusion and emergency surgery. There is an increase in in-hospital mortality in 2019, and this may be due to the revision of the in-hospital mortality definition installed in January 2019 (Supplementary Fig. 5). In the updated format, in-hospital mortality was re-defined as all-cause mortality during hospitalization (or within 30 days of admission) with subclassification into cardiac (procedural and non-procedural), non-cardiac or unknown cause.

**Fig. 4:**
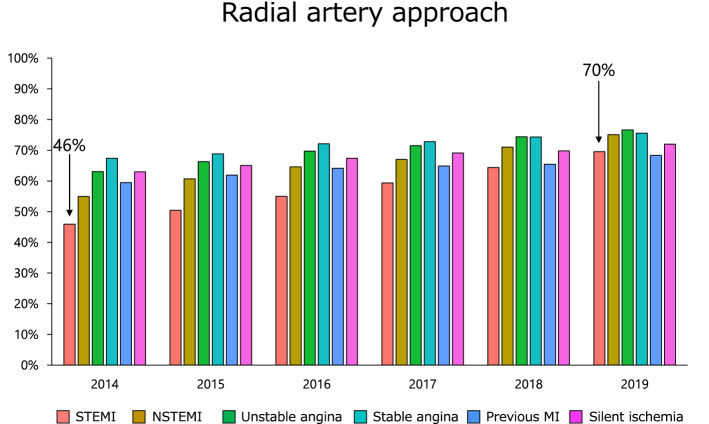
Proportion of radial artery approach. The proportions of PCI with radial artery approach over the past 6 years are shown. Abbreviations as in Fig. 3

**Fig. 5:**
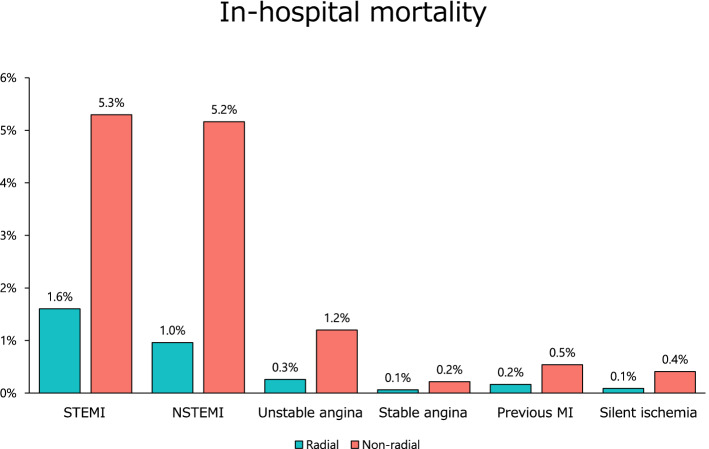
In-hospital mortality with and without radial artery approach. In-hospital mortality according to various clinical presentations with and without radial artery approach is shown. Abbreviations as in Fig. 3

As for the access sites of PCI, the radial artery approach has been recommended in level I class A in both Japanese Circulation Society and European Heart Society clinical practice guidelines [2, 3]. Consequently, there has been a gradual increase in patients with STEMI, from 46% in 2014 to 70% in 2019 (Fig. 4). Moreover, patients that underwent PCI with radial artery had lower crude mortality (Fig. 5) and bleeding complication rate (Supplementary Fig. 6 and Supplementary Fig. 7).

## Discussion

Overall, there was an increase in patient age and the prevalence of dyslipidemia and chronic kidney disease in 2019. While the number of patients with STEMI and NSTEMI increased, that with stable angina showed a downward trend. There was an increase in the percentage of PCI with radial artery approach, which was associated with lower in-hospital mortality and bleeding complications.

The main result of the ISCHEMIA trial (International Study of Comparative Health Effectiveness With Medical and Invasive Approaches) was presented in 2019 and published in 2020 [4]. In this landmark study, an initial invasive strategy, as compared with an initial conservative strategy, did not reduce rates of major cardiovascular events in patients with stable angina, although relief of angina was greater with the invasive approach. The trial results seem largely applicable for the majority of Japanese PCI patients with moderate-to-severe ischemia, and importantly, long-term outcome of the ISCHEMIA-eligible patients were similar to the actual patients enrolled in the trial [5]. Therefore it would be necessary to explore how the publication of trial results has affected the proportion of preprocedural cardiac testing for myocardial ischemia and the number of PCI for stable angina in Japan after 2020.

Furthermore, the outbreak of the novel coronavirus (COVID-19) emerged in late 2019 is the most serious public health threat and has drastically affected medical societies [6]. In response to this confusing situation, CVIT has issued a position statement to its members [7]. Despite the pandemic of COVID-19, nationwide surveys conducted by the CVIT in 2020 reported that most institutions continued to perform primary PCI for the patients presented with STEMI in Japan [8, 9]. In the follow-up studies, we will be able to clarify how the COVID-19 pandemic has affected primary and elective PCI in Japan.

We believe that this large set of data from registered patients analyzed in this survey and collaborative academic projects, will advance research on and treatment outcomes of PCI.

## Supplementary Information

Below is the link to the electronic supplementary material.

Supplementary file1 (DOCX 18 kb)

Supplementary file2 (PDF 735 kb)
