# Supplementary material for: Japanese Nationwide PCI (J-PCI) Registry Annual Report 2019: patient demographics and in-hospital outcomes
Source: Cardiovasc Interv Ther. 2022 Jan 12;37(2):243–7. doi: 10.1007/s12928-021-00832-0 (PMC8753025; doi:10.1007/s12928-021-00832-0)
Supplement: Supplementary file 1 — Supplementary file1 (DOCX 18 kb) [file 12928_2021_832_MOESM1_ESM.docx]

**Supplementary Table 1. Definitions of key baseline variables**

| Definitions of key baseline variables | |
| --- | --- |
| Diabetes | At least one of the following criteria is met: (a) Fasting blood glucose ≥ 126 mg/dL (b) Random blood glucose ≥ 200 mg/dL (c) HbA1c ≥ 6.5 (as per Japanese formula) (d) 2-h 75 g OGTT blood glucose ≥ 200 mg/dL (e) Treatment with oral antidiabetic agents, insulin, or incretin medication |
| Hypertension | At least one of the following criteria should be met based on the Japanese Society of Hypertension 2009 guideline: (a) Systolic blood pressure ≥ 140 mmHg (b) Diastolic blood pressure ≥ 90 mmHg (c) Undergoing treatment with antihypertensive agents |
| Dyslipidemia | Any of the following are met based on the Japan Atherosclerosis Society (JAS) Guidelines for Prevention of Atherosclerotic Cardiovascular Diseases 2012 LDL cholesterol ≥ 140 mg/gL HDL cholesterol < 40 mg/dL Triglycerides ≥ 150 mg/dL LDL cholesterol is calculated using the Friedewald formula (TC–HDL-C–TG/5) (when TG < 400 mg/dL). When TG is ≥ 400 mg/dL or using postprandial blood, non-HDL-C (TC–HDL-C) should be used  *“Fasting” is defined as taking no food for over 10–12 h |
| Smoking | All patients with a history of smoking within the past year |
| Chronic kidney disease | At least one of the following criteria should be met (Japanese Society of Nephrology CKD Treatment Guidelines 2009):  (a) Proteinuria (b) Serum creatinine ≥ 1.3 mg/dL (c) eGFR ≤ 60 ml/min/1.73 m^2^ (eGFR = 194 × age − 0.23 × Cre − 0.1154 [women × 0.742]) |
| Maintenance dialysis | Undergoing hemodialysis or peritoneal dialysis |

HbA1c = Hemoglobin A1c, OGTT = Oral glucose tolerance test, LDL = Low-density lipoprotein cholesterol, HDL = High-density lipoprotein, TG = Triglyceride, CKD = Chronic kidney disease, eGFR = Estimated glomerular filtration rate, Cre = Creatinine

**Supplementary Table 2. Definitions of categories upon clinical presentation**

| Definitions of categories upon clinical presentation | |
| --- | --- |
| Stable angina | Angina with stable symptoms in the past month, with no symptom attacks at rest (symptoms only elicited during high exertion, with no changes in frequency or intensity in the past month) |
| Unstable angina | At least one of the following is met: 1) New-onset angina: Angina, which manifested within the past month 2) Increasing angina: angina that worsened within the past month 3) Resting angina: persistent angina at rest or angina that markedly restricts daily life (symptoms triggered by walking tens of meters or one flight of stairs) 4) Postinfarction angina: persistent angina within 1 month following a myocardial infarction event with the involvement of elevated ST segments on ECG or cardiac biomarkers; if they are, the angina is defined as STEMI or NSTEMI, respectively |
| Acute myocardial infarction | Persistent myocardial ischemia symptoms accompanied by elevated cardiac markers. Elevated cardiac biomarkers refers to elevated creatine kinase (CK) or CK-MB levels [two-folds higher than the normal values] or elevated troponin levels [≥ 99th percentile]  Acute myocardial infarctions are classified as STEMI or NSTEMI as described below: 1) ST-elevation myocardial infarction (STEMI): ST elevation on two or more contiguous leads (≥ 0.2 mV in a precordial lead at the J point or ≥ 0.1 mV in a limb lead), new left bundle branch block, or posterior myocardial infarction on a 12-lead ECG. 2) Non-ST-elevation myocardial infarction (NSTEMI): ECG changes either do not qualify as ST elevation or are not present at all |
| Stent thrombosis | Definite stent thrombosis as defined by the Academic Research Consortium (ARC) (described below).  1. Angiographic confirmation of stent thrombosis The presence of a thrombus that originates from the stent or the segment 5 mm proximal or distal to the stent, and the presence of at least one of the following criteria within a 48-h period:  1) Acute onset of ischemic symptoms at rest 2) New ischemic ECG changes indicative of acute ischemia 3) Typical rise and fall in cardiac biomarkers  2. Pathological confirmation of stent thrombosis Evidence of recent thrombus within the stent at autopsy or by examination of tissue retrieved following thrombectomy |
| Previous myocardial infarction | At least one of the following is met: 1) New abnormal Q wave on an ECG in two or more contiguous leads without evident chest symptoms  2) Confirmation of segmental non-viable myocardium in imaging tests without evident chest symptoms |
| Silent ischemic myocardial infarction | Confirmation of ischemia on a stress ECG or imaging tests (SPECT, stress TTE, stress MRI, etc.) without evident chest symptoms in the past month |

ECG = Electrocardiogram, STEMI = ST-Elevation Myocardial Infarction, NSTEMI = Non-ST-Elevation Myocardial Infarction, CK-MB = Creatine kinase-MB, SPECT = Single-photon emission computed tomography, TTE = Transthoracic echocardiogram, MRI = Magnetic resonance imaging
