# Supplementary material for: Japanese Nationwide PCI (J-PCI) Registry Annual Report 2019: patient demographics and in-hospital outcomes
Source: Cardiovasc Interv Ther. 2022 Jan 12;37(2):243–7. doi: 10.1007/s12928-021-00832-0 (PMC8753025; doi:10.1007/s12928-021-00832-0)
Supplement: Supplementary file 2 — Supplementary file2 (PDF 735 kb) [file 12928_2021_832_MOESM2_ESM.pdf]

Supplementary figures

Supplementary Figure 1. Average age of registered patients

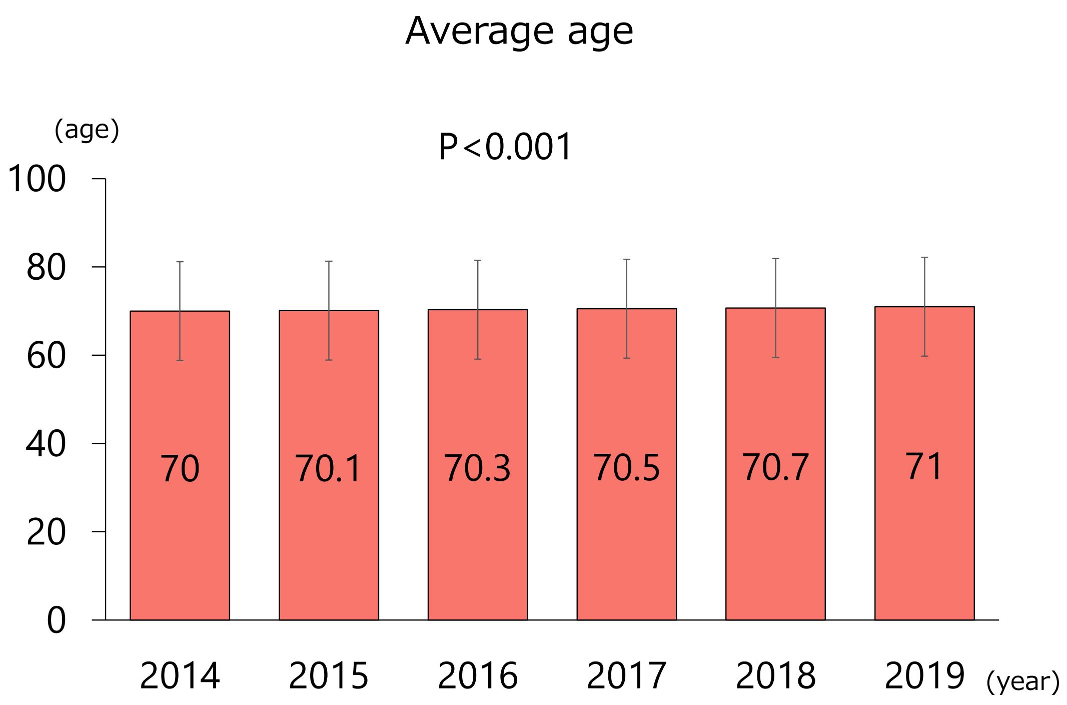

The average ages of registered patients over the past six years are shown.

Supplementary Figure 2. Prevalence of risk factors in 2019

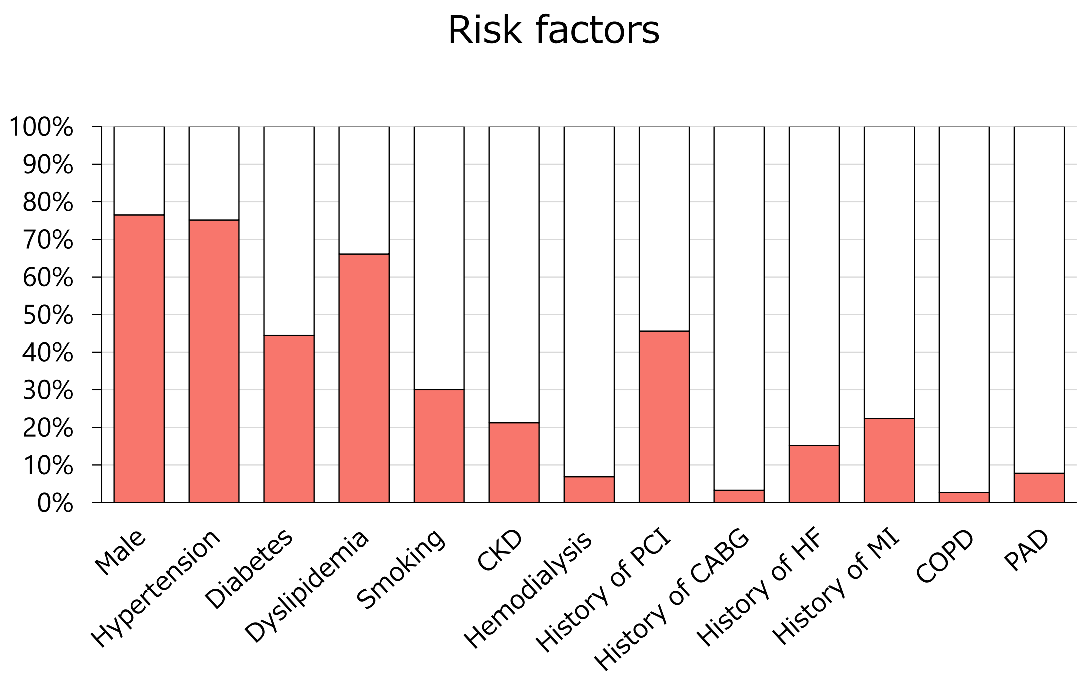

The prevalence of risk factors in2019 is shown.

CKD = chronic kidney disease, PCI = percutaneous coronary intervention, CABG = coronary artery bypass grafting, HF = heart failure, MI = myocardial infarction, COPD = chronic obstructive pulmonary disease, PAD = peripheral artery disease.

Supplementary Figure 3. Number of patients with various clinical presentations in 2019

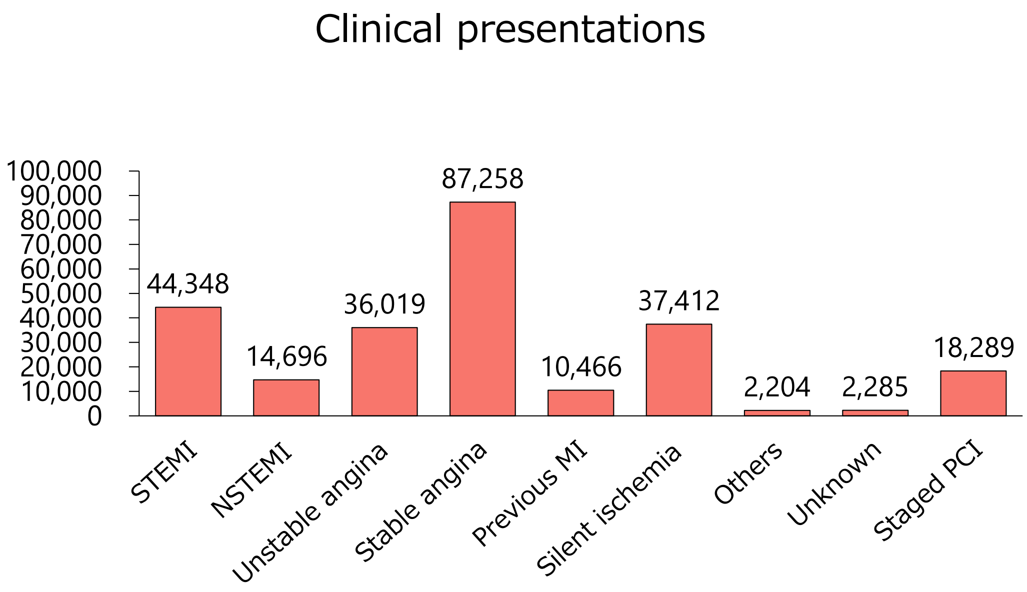

The numbers of patients with various clinical presentations in 2019 are shown.

STEMI = ST-elevation myocardial infarction, NSTEMI = non-ST-elevation myocardial infarction, MI = myocardial infarction, PCI = percutaneous coronary intervention.

Supplementary Figure 4. Number of preprocedural cardiac testing for myocardial ischemia

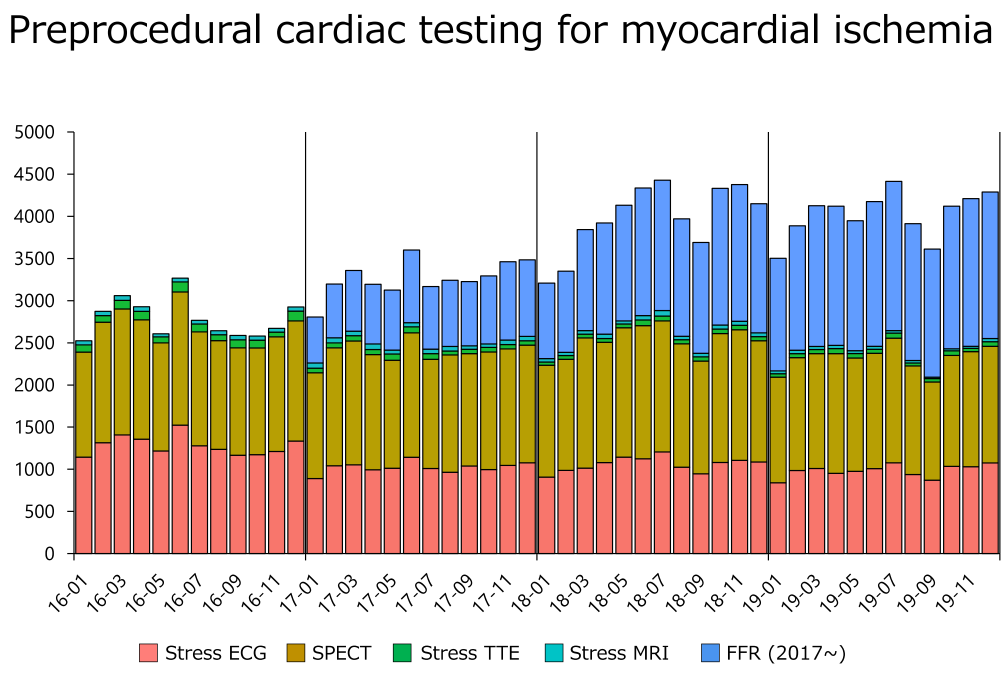

The annual numbers of preprocedural cardiac testing for myocardial ischemia in patients with stable angina over the past four years are shown.

ECG = electrocardiogram, SPECT = single-photon emission computed tomography, TTE = transthoracic echocardiography, MRI = magnetic resonance imaging, FFR = fractional flow reserve.

Supplementary Figure 5. Number of in-hospital outcomes

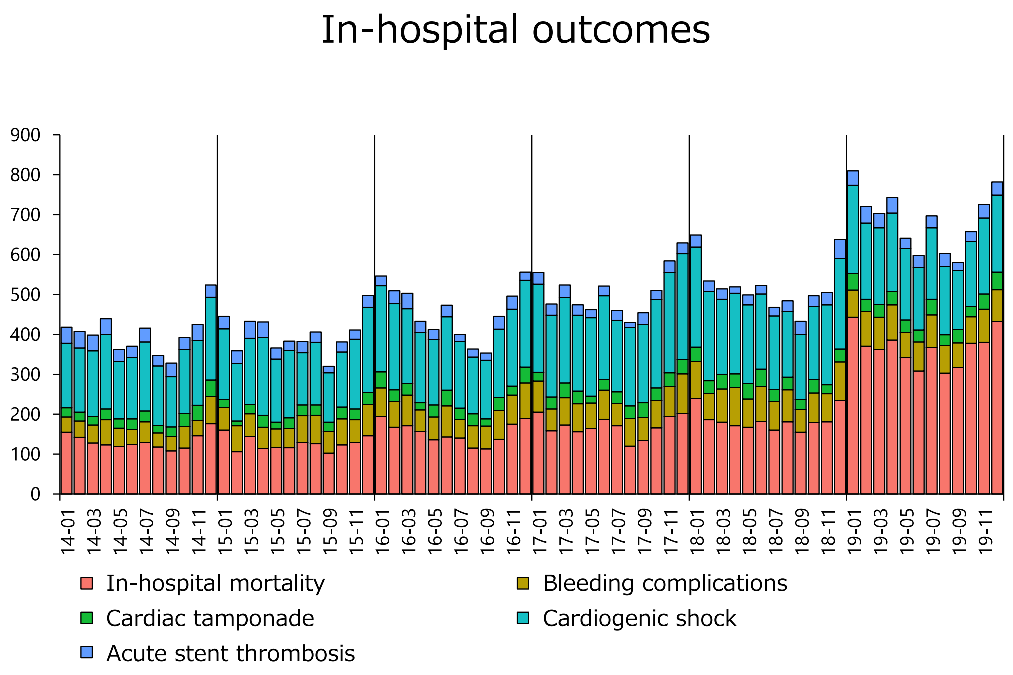

The annual numbers of in-hospital outcomes over the past six years are shown.

Supplementary Figure 6. Frequency of bleeding complications with and without radial artery approach

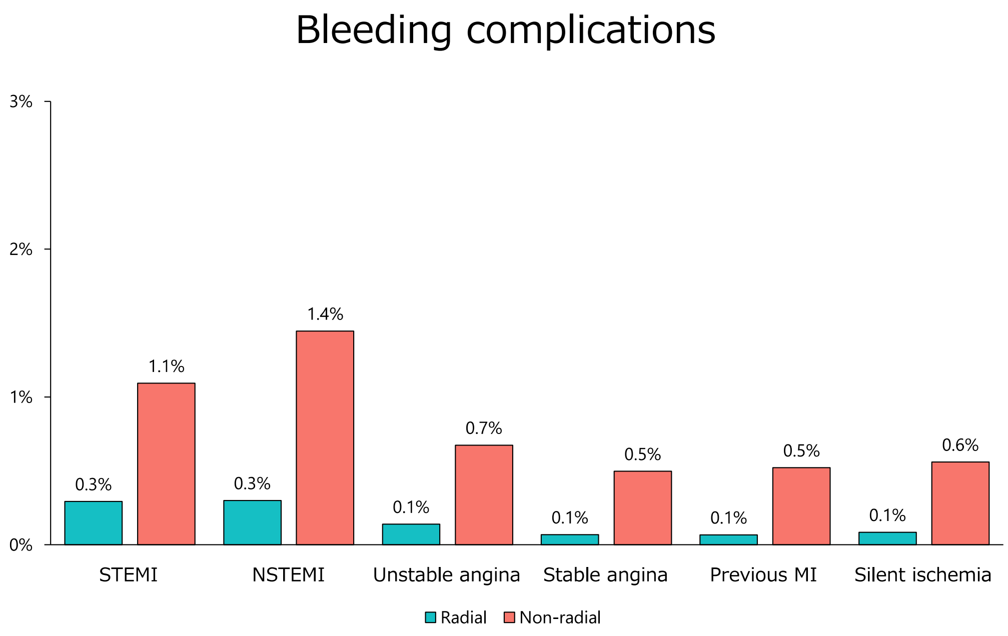

Frequency of bleeding complications according to various clinical presentations with and without radial artery approach is shown.

Abbreviations as in Supplementary Figure 3.

Supplementary Figure 7. Frequency of bleeding complications from access site with and without radial artery approach

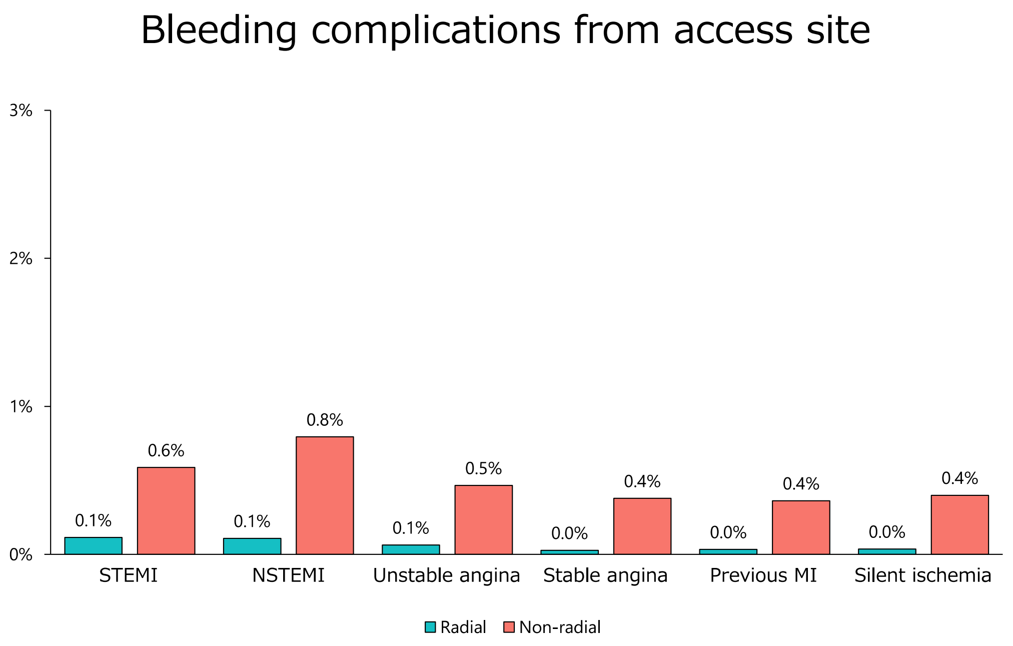

Frequency of bleeding complications from access site according to various clinical presentations with and without radial artery approach is shown.

Abbreviations as in Supplementary Figure 3.
